# Supplementary material for: Are carbon emissions trading and green financial instruments synergistic? -Comprehensive quantitative research based on content analysis
Source: PLoS One. 2024 Mar 7;19(3):e0298601. doi: 10.1371/journal.pone.0298601 (PMC10919601; doi:10.1371/journal.pone.0298601)
Supplement: S1 Data — (ZIP) [file pone.0298601.s001.zip › Data/地级市Z_F工作报告.zip!/╜¡╦╒/╦╒╓▌╩╨/└┤╫╘╣1⁄2╓┌║┼í╛┬φ┐╦╩2╛▌═°í┐_2016.docx]

各位代表：

    现在，我代表苏州市人民政府，向大会报告工作，请予审议，并请市政协各位委员和其他列席人员提出意见。

一、2015年工作回顾

　　去年，面对宏观环境的复杂变化，特别是经济下行压力加大的严峻挑战，我们在中共苏州市委的正确领导下，认真学习贯彻党的十八大、十八届三中四中五中全会和习近平总书记系列重要讲话特别是视察江苏重要讲话精神，牢牢把握“四个全面”战略布局，团结依靠全市人民，主动适应经济发展新常态，统筹抓好稳增长、促改革、调结构、重生态、惠民生、防风险等各项工作，较好地完成了市十五届人大四次会议确定的目标任务。这一年里，第四次中国-中东欧国家领导人会晤、第53届世界乒乓球锦标赛和国际能源变革论坛等重大国际活动在我市成功举办，苏州工业园区获国务院批准在全国首个开展开放创新综合试验，全面提升了苏州的知名度、美誉度和竞争力、影响力。

　　1.经济质量效益稳步提高。全市预计完经济质量效益稳步提高。成地区生产总值1.45万亿元，比上年增长7.5%；一般公共预算收入1560.8亿元，增长8.1%；社会消费品零售总额4424.8亿元，增长9%；全社会固定资产投资6124亿元。积极对接中国制造2025，大力发展新兴产业，促进产业跨界融合，加快产业转型升级。实现规模以上工业总产值3.05万亿元；新兴产业产值增长2.2%，占规模以上工业总产值比重达到48.7%。服务业快速发展，实现增加值7170亿元，增长9%，占地区生产总值比重达到49.5%，形成“三二一”产业发展格局。苏州工业园区和昆山花桥国家现代服务业综合试点积极推进。新增各类金融机构41家，金融总资产增长11.8%；苏州金融资产交易中心获批开业。电子商务示范城市建设成效明显，电子商务交易额达到7000亿元，增长40%；昆山海峡两岸电子商务经济合作实验区获批设立。实现旅游总收入1885亿元，增长11%；我市成为中国国际旅游特色示范城市，苏州高新区镇湖生态旅游区获批国家生态旅游示范区，苏州工业园区阳澄湖半岛旅游度假区升格为国家级旅游度假区。文化产业主营业务收入达到4100亿元，增长15%。第四届中国苏州文化创意设计产业交易博览会成功举办。体育产业快速发展，我市被确定为全国体育产业联系点城市。农业现代化加快推进，新增市级以上现代农业园区12个，新建高标准农田5.33千公顷，吴江区获批国家现代农业示范区。全国质量强市示范城市创建工作扎实开展，新增2家国家品牌培育示范企业，全国首个新兴产业标准化协作平台在我市成立。

　　苏南国家自主创新示范区核心区建设大力推进，区域创新体系逐步完善。落实研发费用加计扣除等政策，减免企业所得税77.4亿元，增长16.1%。全社会研究与试验发展经费支出占地区生产总值比重达到2.68%。新认定国家高新技术企业712家，高新技术产业产值增长2.7%，占规模以上工业总产值比重达到45.9%。新增国家企业技术中心5家、国家地方联合工程中心1家、省级工程中心（工程实验室）9家。我市与清华大学签署创新行动计划合作协议，与北京大学合作共建独墅湖创业大学，中科院上海硅酸盐研究所太仓园区开工建设。国家技术转移苏南中心作用进一步发挥，中科院科技服务网络和国家信息中心软件评测苏州中心启动运营。知识产权强市加快建设，万人发明专利拥有量达到27.4件，比上年增加8.9件；新增驰名商标15件，累计达到112件；张家港市成为国家知识产权示范城市和版权示范城市。科技与金融结合持续深化，科技信贷省、市、县三级联动机制不断完善，成立全国首个科技保险创业投资基金。实施科技创业天使计划和创客天堂行动，国家级、省级新型孵化机构分别达到8家和47家，均名列全省第一。国际精英创业周、“赢在苏州”海外系列创业大赛、苏州技能英才周取得新成果，全市人才总量达到227万人。新增国家“千人计划”人才30人，累计达到187人，其中创业类人才107人，继续位居全国城市首位。

　　2.发展动力活力持续增强。政府机构改发展动力活力持续增强。革深入推进，卫生计生部门完成合并，工商、质监、食药监部门的食品监管职能实施归并，不动产登记机构整合完毕。经济发达镇行政管理体制改革和“区政合一”管理模式试点成效明显。取消、下放行政审批事项9项，事中事后监管得到加强。苏州工业园区获批相对集中行政许可权改革国家试点，姑苏区、苏州高新区获批省级试点。深化预算管理制度改革，强化政府性债务管理，实行政府预算全口径公开。市属行业协会、商会与行政机关脱钩工作基本完成。事业单位分类改革有序开展。以管资本为主加强国有资产监管，资产规模和企业效益实现重大突破。新增上市公司8家、“新三板”挂牌企业158家。落实注册资本实缴认缴、先照后证等举措，在全省率先实施“三证合一”、“一照一码”、“全程电子化”登记制度改革。新增私营企业6.5万家，累计达到35.7万家；新增注册资本2548亿元，增长69.2%。新增跨国公司总部外汇资金集中运营试点企业10家，张家港保税区外债宏观审慎管理试点启动实施。

　　对外贸易保持稳定，完成进出口总额3053.5亿美元，其中出口1814.6亿美元。外贸结构不断优化，一般贸易比重继续提高，加工贸易转型升级步伐加快，服务贸易实现稳步增长。实际使用外资70.2亿美元，其中服务业、战略性新兴产业和高技术项目使用外资分别占到38.1%和48.2%。昆山深化两岸产业合作试验区改革创新领域不断拓展，常熟高新技术产业开发区升格为国家级高新区，张家港、吴中省级高新区获批设立，常熟、吴江、吴中三家出口加工区转型为综合保税区。太仓港成为全省首个国际贸易“单一窗口”试点口岸，与上海港实现通关通检和物流一体化。“走出去”步伐加快，境外实际投资额超过16亿美元，增长116%，实现全省“十二连冠”。“苏满欧”五定班列实现常态化运行，首个国家级对外投资服务示范平台落户苏州工业园区，埃塞俄比亚东方工业园被确定为国家级加工制造型境外经贸合作区。南北共建合作成效显著，对口支援和帮扶工作积极开展。外事、对台、侨务工作全面深化，新增国际友城1个、全国社区侨务工作示范单位2家。

　　3.城乡一体发展加快推进。中心城区城城乡一体发展加快推进。市开发边界划定工作基本完成，制定市区镇村布局规划，完善古城保护控制性详细规划和阊门历史文化街区保护规划。沪通铁路苏州段建设有序推进。常嘉高速公路、张家港疏港高速公路抓紧建设，312国道苏州西段改扩建顺利完工。西环快速路北延和东环快速路南延一期工程建成通车，一批市政道路相继建成。轨道交通2号线延伸线、4号线及支线完成轨道铺设，3号线开工建设。我市入选国家首批地下综合管廊试点城市。新增人防设施120万平方米。苏州港货物吞吐量达到5.4亿吨，增长13.4%；其中太仓港集装箱运量超过370万标箱，增长21.2%。苏南运河苏州段四级航道改三级航道和杨林塘航道整治基本完成。七浦塘拓浚整治工程全线通水。1000千伏特高压淮上线工程和智能电网应用先行区、示范区加快建设。“千兆苏州”全光网顺利建成，市域地理国情普查通过验收。深入开展“共建美丽新苏州、同享美好新生活”城市环境综合整治提升行动和“城市环境大家管、市容秩序大提升”治理活动，市容市貌得到改善。

　　城乡发展一体化综合改革纵深推进，农村发展更具活力。新型城镇化与城乡发展一体化规划制定出台。农村土地承包经营权确权登记颁证工作全面开展，农村产权交易试点顺利推进。新增社区股份合作社股权固化改革试点村（社区）369个、“政经分离”改革试点村（社区）100个，吴中区被列为全国农村集体资产股份权能改革试点。新增各类农村合作社123家，累计达到4535家。农村集体经济继续壮大，集体资产达到1610亿元；村均收入达到776万元，增长8.1%。新建“一村二楼宇”物业载体113.8万平方米。设立全省首个城乡一体化基金，财政金融服务“三农”发展的能力明显增强。完成美丽城镇建设项目333个，建成美丽村庄示范点10个、三星级康居乡村100个。启动实施农村生活污水治理三年行动计划，完成1000个自然村庄治理任务，我市被确定为全国村庄生活污水治理试点市，常熟市被确定为全国县域村镇污水治理综合示范区。

　　4.生态环境质量继续改善。切实保障饮生态环境质量继续改善。用水安全，集中式饮用水水源地水质达标率保持100%。实施太湖流域水污染治理项目630个，完成投资44.1亿元。阳澄湖生态优化行动持续开展，湖体水质得到提升。加大古城河道治理力度，基本完成背街水巷污水排放综合整治任务。大力实施大气污染治理项目，加快淘汰燃煤小锅炉、黄标车和老旧机动车，PM　年均浓度下降12.1%，市区环境空气质量达标天数为240天，比上年增加13天。认真开展噪声污染防治、重金属污染整治、土壤污染调查和修复工作，强化核与辐射环境监管，全面规范危险废弃物管理。加强农业面源污染治理，农业废弃物无害化处理及资源化利用率达到96.3%。严把环保关口，坚决拒批不符合环保要求的项目，关停淘汰落后企业1116家。推进国家循环经济示范城市和低碳试点城市建设，做好园区循环化改造示范试点工作，单位地区生产总值能耗下降、主要污染物排放总量削减完成省下达任务。

　　制定生态红线区域保护配套制度、生态文明建设考核办法和生态补偿条例实施细则，探索建立环境资源限额分配、有偿转让制度。实施生态文明建设重点工程95项，完成投资180亿元。吴中区被列为国家级生态保护与建设示范区。上方山石湖生态园南区项目基本建成，七子山垃圾填埋场生态修复主体工程按期竣工。市区新增绿地430万平方米、农村新增林地绿地768公顷，全市陆地森林覆盖率达到29.56%，森林防火措施进一步完善。露采矿山环境整治年度任务全面完成。苏州市和昆山市创建国家生态园林城市通过考评。

　　5.社会建设治理全面加强。优化教育设社会建设治理全面加强。

　　施布局，新建公办中小学、幼儿园41所。基础教育课程和课堂教学改革不断深化，中小学学业质量阳光指标评价系统初步建立。现代职教体系衔接试点工作有序开展，校企合作深入推进。省市共建力度进一步加大，在苏高校办学水平全面提升，苏州科技学院更名苏州科技大学通过专家评审。世界联合学院常熟分校招生开学。民办教育加快发展，建成一批民办学校。“学在苏州”市民终身学习云平台开通运行。推进省医药卫生体制改革试点和国家公立医院改革试点工作，在全省率先实现公立医院药品零差率销售。我市通过国家卫生城市复审。苏州大学附属第一医院平江新院和附属儿童医院园区总院投入使用，苏州科技城医院基本建成。智慧医疗卫生信息化、公益性应急救护培训、母婴阳光工程和婴幼儿健康促进工程持续开展。公共文化服务体系进一步完善，新增市图书馆分馆10家，市档案馆新馆、苏州现代传媒广场和妇女儿童活动中心迁建工程如期建成，苏州高新区文体中心主体完工。融资规模超过百亿元的华人文化控股基金落户苏州工业园区。“16+1”文艺演出获李克强总理批示肯定。第六届中国昆剧艺术节、第十四届中国戏剧节、第六届中国苏州评弹艺术节成功举办。加强历史文化名城保护，平江历史文化片区成片保护利用、虎丘地区综合改造、桃花坞历史文化片区保护整治和古建老宅保护修缮等工程稳步推进。可园（一期）等古典园林修复开放，首批苏州园林名录向社会公布。江南水乡古镇和张家港黄泗浦遗址、太仓浏河天妃宫遗迹申报世界文化遗产工作积极开展。省级公共体育服务体系示范区建设任务全面完成，环古城河、石湖景区健身步道建成开放，苏州工业园区体育中心建设进展顺利。张家港市入选全国青少年校园足球试点县（区）。在全省率先完成县级市、区地方志二轮编纂工作。少数民族群众服务体系不断完善，宗教工作进一步加强。国防动员和民兵预备役工作扎实推进，争创全国双拥模范城“六连冠”工作积极开展，军民融合发展取得新进步。

　　中国特色社会主义和中国梦深入人心，社会主义核心价值观广为弘扬。围绕中国人民抗日战争暨世界反法西斯战争胜利70周年，广泛开展群众性宣传教育系列纪念活动。苏州市、张家港市蝉联全国文明城市。市公民道德馆建成开馆。民主法治建设成效明显，我市公众法治满意度名列全省第一，吴中区、相城区获评全国法治县（市、区）创建活动先进单位。公共法律服务体系基本覆盖到村，法律援助范围进一步扩大。“三社联动”和社区减负工作继续深化，社区服务功能逐步完善。姑苏区被评为全国社区治理和服务创新实验区，苏州工业园区中新社会治理合作试点取得成效。社会信用体系加快建设，我市成为国家信用建设示范城市。社科强市工程深入实施。创建省级科普示范县（市、区）实现“满堂红”。严格落实安全生产监管责任，制定实施企业主体责任“十项制度”，安全生产责任体系不断健全。深入开展“打非治违”和专项整治行动，隐患排查整改力度持续加大，有效遏制了重特大事故发生。应急管理教育培训积极开展，各类突发事件得到妥善解决。引导群众依法逐级走访，实施诉访分离，做好矛盾纠纷排查化解工作，解决了一批信访积案和疑难复杂纠纷。加强社会治安综合治理，开展新一轮平安苏州建设，圆满完成国家重大活动期间的安保维稳任务，社会保持和谐稳定。

　　6.人民生活水平不断提升。城镇居民人人民生活水平不断提升。均可支配收入达到5.04万元，农村居民人均可支配收入达到2.57万元，分别增长8%和9%。实事项目如期完成。新增就业17万人，开发公益性岗位1万个，苏州籍应届高校毕业生就业率达到98.6%，城镇登记失业率为1.9%。推进大众创业、万众创新，累计建成各类创业基地235家，孵化面积超过520万平方米。城镇职工社会保险覆盖率、城乡居民养老保险和医疗保险覆盖率均保持在99%以上，城乡居民大病保险制度基本建立。退休人员基本养老金平均每人每月增加198.3元，居民社会养老保险基础养老金提高到每人每月200元以上。失业保险金最低标准、城乡最低生活保障标准分别提高到每人每月975元和750元。新增养老机构床位6099张、日间照料中心247个、助餐点114个、助浴点18个。残疾人就业扶持和特困救助力度进一步加大。加强住房保障，建成保障房28275套，新开工建设26427套。新增缴存住房公积金职工73.7万人，职工住房公积金使用额增长2.5倍。加快棚户区改造和城中村（无地队）整治，解放新村危旧房改造项目异地安置住宅基本完工，梅巷片区改造项目住宅楼主体全部封顶。改造更新市区老旧燃气管道171.8公里。市区新购公交车664辆，其中新能源公交车608辆，新辟公交线路28条；市交通运输指挥中心建成启用。中心城区农贸市场标准化建设和改造三年任务基本完成。“粮安工程”建设三年行动计划深入实施，粮食流通市场监管得到加强。居民消费价格总水平涨幅为1.6%。发放物价补贴9000万元，惠及群众30万人。建成食品安全快检中心，强化食品药品安全风险监测，大力开展专项治理，切实保障饮食用药安全。

　　与此同时，政府自身建设进一步加强。聚焦对党忠诚、个人干净、敢于担当，扎实开展“三严三实”专题教育，坚持不懈抓好“四风”问题整治，持续改进政府机关作风。政务服务平台加快整合，服务体系不断健全。坚持依法行政，法治政府建设取得新进展。提请市人大常委会审议地方性法规议案2件，颁布政府规章2件，制定规范性文件7件。重大行政决策事项实行目录化管理和网上公开，法制审查制度、重大决策实施后评估制度和档案管理制度建立健全。建立行政执法和刑事司法衔接机制，实施行政执法公示制度，加强行政复议工作，行政执法和行政复议更加规范。全面深化政务公开，公共资源配置、重大建设项目批准和实施、社会公益事业建设等领域的信息公开工作积极推进。公务用车制度改革全面完成。行政经费支出进一步压缩，因公出国（境）、公务用车购置及运行、公务接待财政拨款支出下降15.7%。审计监督继续强化，重大政策措施落实情况跟踪审计、重大投资项目审计、国有企业审计和领导干部经济责任审计成效明显。市政府自觉接受市人民代表大会及其常委会的法律监督、工作监督和市政协的民主监督，坚持重大事项向市人大常委会报告、向市政协通报，注重发挥各民主党派、工商联、无党派人士和人民团体的参政议政作用。组织实施人大议案2件，办复人大代表建议235件、政协提案371件。

　　各位代表，过去一年的成绩来之不易，凝聚着全市人民的辛劳和智慧，得益于方方面面的支持和奉献。在此，我代表市政府向全市人民，向人大代表、政协委员，向离退休老同志，向各民主党派、工商联和无党派人士，向各人民团体，向驻苏解放军、武警和消防官兵，向国家和省驻苏单位，向参与、支持、关心苏州各项事业发展的海内外朋友，表示衷心的感谢和崇高的敬意！

　　回顾过去的一年，我们也看到，经济社会发展和政府工作仍然存在不少问题，主要是：经济下行压力加大，产业结构不尽合理，创新能力亟待增强；制约经济社会发展的体制机制障碍尚未完全破除，深化改革的任务艰巨繁重；资源环境约束趋紧，生态文明建设需要持续用力；基本公共服务配置仍不够均衡，民生改善还有大量工作要做，加强社会治理和城市管理、维护和谐稳定面临不少新情况新矛盾；法治建设有待强化，公务人员特别是领导干部的能力水平还需进一步提高。对此，我们一定高度重视，采取更为有效的措施切实加以解决。

二、“十三五”规划纲要（草案）的主要内容

　　“十三五”时期，是全面建成小康社会、实现“两个一百年”奋斗目标的第一个百年奋斗目标的决胜阶段。我们深入贯彻落实习近平总书记系列重要讲话特别是视察江苏重要讲话精神，按照党的十八届五中全会和省委十二届十一次全会部署，根据《中共苏州市委关于制定苏州市国民经济和社会发展第十三个五年规划的建议》，在广泛深入调查研究基础上，认真编制了《苏州市国民经济和社会发展第十三个五年规划纲要（草案）》。《纲要（草案）》主要有以下内容：

　　1.“十二五”发展的重大成就和宝贵经验

　　过去的五年，在中共苏州市委的带领下，全市人民凝心聚力，接续奋斗，积极抢抓发展机遇，妥善应对风险挑战，攻克种种艰难险阻，付出巨大艰辛努力，胜利完成了“十二五”规划确定的主要目标任务。经济实力实现新提升。地区生产总值年均增长9.5%，按常住人口计算人均超过2.1万美元；规模以上工业总产值稳居全国城市第2位；一般公共预算收入年均增长11.6%；累计完成全社会固定资产投资2.8万亿元。转型升级创出新成效。现代服务业不断壮大，服务业增加值占地区生产总值比重提高7.9个百分点。新兴产业快速发展，占规模以上工业总产值比重提高19.9个百分点。农业基本现代化综合得分连续位居全省首位。区域创新能力继续增强，全社会研究与试验发展经费支出占地区生产总值比重提高0.34个百分点。改革开放取得新进展。市级行政审批事项目录清单、政府行政权力清单、政府部门责任清单等6份清单向社会公布；新一轮行政审批制度改革开展以来，累计削减行政审批事项371项。国有经济活力增强，民营经济快速发展。金融创新力度加大，苏州工业园区、昆山深化两岸产业合作试验区跨境人民币创新业务试点成效明显。全方位开放布局全面展开，苏州在全国全省开放大格局中的地位和作用更加凸显。对外贸易稳步增长，累计实际使用外资419.1亿美元、境外协议投资额71.8亿美元。城乡一体展现新面貌。实施行政区划重大调整，中心城市首位度进一步提高。基础设施更加完善，轨道交通1号线、2号线和中环快速路等重大项目建成使用。全市综合交通网络总里程达到1.57万公里。城乡发展一体化政策制度框架加快健全，工农互惠、共同繁荣的局面初步形成。生态环境和人居环境持续优化，基本公共服务均等化水平有了较大提高。民生福祉得到新改善。城镇居民、农村居民人均可支配收入年均分别增长10.6%和12%。劳动力就业更加充分，城乡社会保险基本实现全覆盖。教育现代化进程加快，高等教育毛入学率超过68%，新增劳动力平均受教育年限达到15.4年。医疗卫生资源优化配置，城乡社区卫生服务普及率和人口覆盖率均达到100%。文化事业繁荣发展，公益性文化设施覆盖到村。法治建设、平安建设走在全国前列，社会安定有序、团结和谐、充满活力。

　　过去五年的成功实践与重大成就不仅为“十三五”发展奠定了坚实基础，更为今后工作积累了弥足珍贵的经验。一是坚持发展第一要务不动摇。主动把握和积极适应经济发展新常态，以提高质量和效益为中心，遵循规律，因势利导，兴利除弊，着力形成稳中有进、稳中提质的良好态势。二是坚持转方式调结构不动摇。以创新驱动为主引擎，大力发展创新型经济，促进发展动力转换，加快产业转型升级，推动经济发展迈上新台阶。三是坚持深化改革开放不动摇。在国家、省全面深化改革大框架下和开放战略总布局中抢抓机遇、快速行动、勇于实践，积极构建有利于科学发展的体制机制，激发市场主体活力，拓展对内对外开放新空间。四是坚持促进协调发展不动摇。统筹经济社会发展，统筹城乡一体发展，统筹经济建设与生态文明建设，注重解决发展中不平衡、不协调、不可持续问题，努力增强发展整体性。五是坚持增进民生福祉不动摇。紧扣习近平总书记提出的“七个更”的民生工作重点要求，持续加大民生投入力度，扎实办好民生实事，让改革发展成果更多更公平更实在地惠及全市人民。

　　2.“十三五”发展的指导思想和目标定位

　　“十三五”时期全市经济社会发展的指导思想是：高举中国特色社会主义伟大旗帜，全面贯彻党的十八大和十八届三中、四中、五中全会精神，以马克思列宁主义、毛泽东思想、邓小平理论、“三个代表”重要思想、科学发展观为指导，深入贯彻习近平总书记系列重要讲话特别是视察江苏重要讲话精神，紧紧围绕“四个全面”战略布局，牢固树立和贯彻落实创新、协调、绿色、开放、共享的发展理念，坚持发展是第一要务和稳中求进工作总基调，着力加强供给侧结构性改革，以提高发展质量和效益为中心，加快形成引领经济发展新常态的体制机制和发展方式，始终牢记“两个率先”光荣使命，以“五个迈上新台阶”为重点任务，以落实省委、省政府“八项工程”为主抓手，统筹推进经济建设、政治建设、文化建设、社会建设、生态文明建设和党的建设，当好建设“经济强、百姓富、环境美、社会文明程度高”新江苏的先行军排头兵，率先全面建成小康社会，积极探索开启基本实现现代化建设新征程，谱写好中华民族伟大复兴中国梦的苏州篇章。

　　苏州的发展定位是：努力建成具有国际竞争力的先进制造业基地、具有全球影响力的产业科技创新高地、具有独特魅力的国际文化旅游胜地和具有较强综合实力的国际化大城市。

　　“十三五”发展的具体目标是：

　　——经济保持中高速增长、产业迈向中高端水平，综合实力与核心竞争力显著提升。地区生产总值年均增长7%左右，提前完成比2010年翻一番的目标；服务业增加值占地区生产总值比重达到53%左右，高新技术产业产值占规模以上工业总产值比重达到50%，现代农业发展水平指数达到92；全社会研究与试验发展经费支出占地区生产总值比重达到3%左右，科技进步贡献率超过65%，万人发明专利拥有量达到36件；高层次人才总量达到20万人。

　　——居民收入增长和经济增长同步、劳动报酬提高和劳动生产率提高同步，提前完成城乡居民人均收入比2010年翻一番的目标，城乡居民收入差距进一步缩小；城镇调查失业率为5%左右；城乡基本社会保险覆盖率保持在99%以上；人均预期寿命达到83岁。

　　——单位地区生产总值能耗下降、主要污染物排放总量削减完成省下达任务；单位地区生产总值建设用地占用降低到每亿元13.7公顷左右；陆地森林覆盖率达到30%；空气、水和土壤污染治理取得明显成效，城乡生态环境和人居环境不断优化。

　　——高等教育毛入学率达到70%；每千人拥有执业医生2.5人以上；基本建成城乡10分钟文化圈；人均公共体育场地面积达到3.2平方米；人民群众对法治建设的满意率超过92%；城乡和谐社区建设达标率超过95%。

　　3.“十三五”发展的基本理念

　　实现“十三五”发展目标，必须自觉践行五大发展理念。

　　一是自觉践行创新发展理念。把创新摆在苏州发展全局的核心位置，大力实施创新驱动发展战略和人才强市战略，不断推进经济社会各领域创新，全面深化改革，培育发展新动力，拓展发展新空间，构建产业新体系，营造有利于科技创新、人才成长和大众创业、万众创新的良好环境，为引领经济发展新常态、实现产业转型升级提供坚实支撑和强劲动力。

　　二是自觉践行协调发展理念。牢牢把握中国特色社会主义事业总体布局，正确处理发展中的重大关系，大力实施区域联动发展战略、城乡发展一体化战略和空间优化战略，重点促进城乡区域协调发展，促进物质文明与精神文明协调发展，促进经济社会协调发展，促进新型工业化、信息化、城镇化、农业现代化同步发展，推动硬实力和软实力同步提升。

　　三是自觉践行绿色发展理念。坚持生态优先，全面贯彻节约资源和保护环境的基本国策，大力实施可持续发展战略，统筹推进生态环境保护、生态经济发展和生态文明制度建设，实现经济社会发展与生态环境改善相互协调、相互促进，加快建设资源节约型、环境友好型社会。

　　四是自觉践行开放发展理念。顺应苏州经济深度融入世界经济的趋势，大力实施开放提升战略，坚持内外需协调、进出口平衡、更高水平“引进来”和更大步伐“走出去”并重、引资和引技引智并举，丰富开放内涵，提高开放水平，建立对外开放新体制，形成更高层次、更宽领域、互利共赢的开放格局。

　　五是自觉践行共享发展理念。坚持人民主体地位，按照人人参与、人人尽力、人人享有的要求，大力实施民生共建共享战略，认真落实为民办实事长效机制，持续增加城乡居民收入，全面提高基本公共服务均等化水平，维护社会公平正义，促进人的全面发展，巩固和谐稳定的大好局面。

　　4.“十三五”发展的主要任务

　　《纲要（草案）》提出，“十三五”时期要建立具有苏州特点的十大体系。这十大体系紧紧围绕“经济强、百姓富、环境美、社会文明程度高”的总要求，对今后五年的发展任务作了具体安排。

　　一是以质量效益为中心推进“经济强”。《纲要（草案）》提出建立具有综合竞争优势的产业科技创新体系、双轮驱动的现代产业体系、双向互动的开放型经济体系、多规融合的区域增长空间体系。具体来说，在动力上，强调发展的基点在于创新，突出科技创新在全面创新中的引领作用，注重依靠创新汇聚融合高端要素，加快实现发展动力转换。在产业上，强调抓住转方式、调结构这一关键，坚持调高、调轻、调优、调强、调绿的导向，做强先进制造业和现代服务业两大主干，推动现代农业建设迈上新台阶，实现产业优化升级。在开放上，强调扩大双向开放，放大向东开放优势，做好向西开放文章，更好利用国际国内两个市场、两种资源，坚定不移推进企业、城市、人才国际化，发展更高层次的开放型经济。在空间上，强调优化区域发展布局，培育形成新的战略增长极，提升整体发展水平。

　　二是以普惠共享为目标推动“百姓富”。《纲要（草案）》提出建立普惠共享的社会发展体系，并对如何提高人民生活水平和质量作出了具体规划。强调促进就业创业，构建更加公平更可持续的社会保障制度，健全覆盖城乡的基本公共服务体系，增强公共产品和公共服务供给能力，更好满足人民群众需求。实现“百姓富”的重点和难点在农村。《纲要（草案）》提出建立均衡协调的城乡一体化发展体系，强调深化城乡发展一体化综合改革，破除城乡二元结构，让城乡全体人民共同迈入全面小康社会。

　　三是以绿色宜居为导向营造“环境美”。《纲要（草案）》提出建立生态宜居的绿色发展体系和功能完善的特大型城市运行管理体系。强调坚定走生产发展、生活富裕、生态良好的文明发展道路，更大力度推进生产方式、生活方式、消费方式绿色化，加快建设主体功能区，筑牢生态安全屏障，促进人与自然和谐共生。主动适应特大城市发展趋势，提高基础设施建设水平，优化环境面貌，加强古城保护，传承历史文化，提升城市品位，使苏州自然环境之美、景观风貌之美、品质特色之美更加突出和鲜明。

　　四是以和谐向上为追求促进“社会文明程度高”。《纲要（草案）》提出建立特色鲜明的软实力发展体系和多元主体参与的现代社会治理体系。强调加强先进思想文化引领，培育和践行社会主义核心价值观，弘扬诚实守信的社会风尚，促进社会主义文化大发展大繁荣，为改革发展提供坚强思想保证、强大精神动力、丰富道德滋养。加快建设法治经济、法治社会、法治政府，在全社会形成浓厚的法治氛围。加强和创新社会治理，推进社会治理精细化，构建社会共治格局。

三、2016年主要任务

　　根据中共苏州市委十一届十次全体（扩大）会议的部署，今年政府工作的总体要求是：以习近平总书记系列重要讲话特别是视察江苏重要讲话精神为引领，全面贯彻党的十八大、十八届三中四中五中全会和中央、全省经济工作会议精神，按照“五位一体”总体布局和“四个全面”战略布局，认真践行五大发展理念，主动适应经济发展新常态，坚持稳中求进工作总基调，积极落实宏观政策要稳、产业政策要准、微观政策要活、改革政策要实、社会政策要托底的要求，着力加强供给侧结构性改革，在适度扩大总需求的同时，去产能、去库存、去杠杆、降成本、补短板，切实提高供给体系质量和效率，突出抓好稳定增长、转型创新、改革开放、城乡建设、生态优化、民生改善等重点工作，全力推动经济平稳健康发展与社会和谐进步，确保实现“十三五”发展的良好开局。

　　全市经济社会发展的主要预期目标是：地区生产总值增长7.5~8%，一般公共预算收入增长8%左右，全社会固定资产投资6000亿元左右，社会消费品零售总额增长8.5%左右，进出口总额保持稳定，实际使用外资60亿美元左右，城乡居民人均可支配收入增幅高于地区生产总值增幅，居民消费价格总水平涨幅控制在3%左右，城镇调查失业率控制在3.5%以内，全社会研究与试验发展经费支出占地区生产总值比重超过2.7%，单位地区生产总值能耗下降、主要污染物排放总量削减完成省下达任务。

　　在新的一年里，我们将重点做好以下八个方面工作。

　　1.优化供需结构，力促经济平稳增长。优化供需结构，力促经济平稳增长。积极运用市场机制、经济手段、法治办法，持续化解过剩产能，对资不抵债、扭亏无望的企业加快兼并重组或依法破产，对高耗能、高污染企业坚决予以关停淘汰，并妥善分流安置企业职工。按照加快提高户籍人口城镇化率和深化住房制度改革的要求，加强监测调控，注重供需对接，有效释放刚性需求和改善性需求，促进房地产市场稳定健康可持续发展。加强对实体经济的服务支持、财税支持和信贷支持，开展降低实体经济企业成本行动，优化企业发展环境，使优质企业增强竞争力，为困难企业松绑助力。鼓励和支持各种所有制企业创新发展，培育壮大规模型骨干企业，促进中小微企业快速成长，增强企业家信心，激发市场主体的积极性和创造性。督促和指导企业提高权益资本比重、降低负债率。开展金融风险专项整治，加强金融监管，规范融资行为。严控政府新增债务，做好存量债务置换工作。加快补齐城乡基础设施建设、民生保障、公共服务等方面的短板，增强发展的均衡性。

　　积极调整需求结构，进一步释放新需求。发挥投资对增长的关键作用，增加有效投入，集中力量建设总投资超过1万亿元的200项重大项目，年内完成投资1500亿元以上。优化投资结构，重点布局和实施一批新兴产业、现代服务业项目。引导社会资本投向政府鼓励项目和符合国家产业政策的领域，推广政府和社会资本合作模式。超前谋划、储备一批战略性项目，增强投资后劲。发挥消费对增长的基础作用，以扩大服务消费为重点带动消费结构升级，引导消费朝着智能、绿色、健康、安全方向转变。加快国家电子商务示范城市建设，推动商贸流通企业线上线下融合发展。实施全域旅游战略，打造精品旅游产品和创意项目，注重发展古城旅游和乡村旅游，做大做强旅游经济。发展新型文化业态，扩大文化消费。发挥出口对增长的促进作用，实施以质取胜、品牌国际化、市场多元化战略和产业链提升工程，提高出口商品竞争力，全力稳定外贸增长。引导加工贸易向价值链中高端攀升，促进一般贸易向自有品牌、自主创新产品提升，推动外贸向优质优价、优进优出转变。积极发展服务贸易，保持服务外包业务平稳增长。

　　2.强化创新驱动，提升产业发展层次。强化创新驱动，提升产业发展层次。深入实施创新驱动发展战略，依靠科技进步提高全要素生产率。加快建设苏南国家自主创新示范区核心区，推动苏州工业园区争创世界一流高科技产业园区，苏州高新区跻身全国高新区第一方阵，昆山、常熟高新区建设创新型特色园区，力争汾湖高新技术产业开发区升格为国家级高新区，太仓、吴江高新技术产业开发区成为省级高新区。强化企业创新主体地位和主导作用，壮大以高新技术企业为骨干的创新型企业集群，高新技术产业产值占规模以上工业总产值比重提高1个百分点。落实“雏鹰”、“瞪羚”计划，扶持科技型中小企业发展。全面推进金融支持企业自主创新行动计划，充分发挥创业投资、天使投资的重要作用，创新“科贷通”产品体系，推动产业链、创新链和资金链有效对接。深化与科研院所、著名高校的合作，争取更多重大科研机构落户我市。构建产业技术创新联盟，促进跨领域跨区域协同创新。启动苏州自主创新广场二期建设，完善科技服务载体功能。深入实施创客天堂行动，加快形成金鸡湖创业长廊、创客峰汇等众创服务集聚区。探索知识产权综合管理改革，加强知识产权运用和保护，建设江苏（国际）知识产权运营交易中心。实施技术标准化战略，建设质量强市和品牌强市。落实各项人才计划，精心举办国际精英创业周、“赢在苏州”海外系列创业大赛等活动，大力培养集聚复合型创新创业人才、高科技高技能领军人才和战略性新兴产业高端人才。

　　落实中国制造2025苏州实施纲要，推动制造业向高新化、品牌化、服务化、智能化发展。培育壮大新一代电子信息、高端装备制造、新材料、软件和集成电路、新能源和节能环保、医疗器械和生物医药等新兴产业，力争新兴产业产值占规模以上工业总产值比重提高1个百分点。开展智能装备、智能制造专项行动，建设智能车间、智能工厂。改造提升传统产业，全面提高产品技术、工艺装备、能效环保等水平。加强特色产业基地建设，形成一批优势产业链和产业集群。引导中小企业走“专精特新”之路。促进服务业优质高效发展，推动生产性服务业向专业化和价值链高端延伸、生活性服务业向便利化精细化品质化拓展、制造业由生产型向生产服务型转变，力争服务业增加值占地区生产总值比重提高1个百分点。加快现代服务业集聚区提档升级，着力引进和培育总部企业。认真落实“互联网+”行动计划，促进互联网和经济社会融合发展。实施大数据战略，推进数据资源开放共享。坚持走产出高效、产品安全、资源节约、环境友好的农业现代化道路，提高农业质量和效益。发展合作组织、家庭农场、专业大户、龙头企业等农业经营主体，培养新型职业农民。开展新一轮高标准农田建设，新增现代农业园区面积4.67千公顷、高标准农田3.33千公顷。创新农产品产销对接模式，引导和支持网上销售。

　　3.深化改革开放，增创体制机制优势。深化改革开放，增创体制机制优势。坚持中国特色社会主义政治经济学的重大原则，坚持社会主义市场经济改革方向，使市场在资源配置中起决定性作用。围绕简政放权、放管结合、优化服务，深化行政审批制度改革，严格实行清单管理，强化事中事后监管。规范行政审批中介服务，促使中介组织回归市场定位和服务本位。深入开展经济发达镇行政管理体制改革，启动第二批试点镇改革工作，重点推动综合执法和便民服务规范化、标准化。加大商事制度改革力度，推行全程电子化登记，加快工商注册便利化。分类推进国有企业改革，完善现代企业制度。加强经营性国有资产管理，确保国有资产保值增值。鼓励民营企业依法进入更多领域，引入非国有资本参与国有企业改革。推进金融创新，发展普惠金融。建设环境能源交易中心和基金产业集聚区，建成苏州股权交易中心（江苏股权交易中心苏州分中心）。推动跨境电子商务、跨境人民币交易、外债宏观审慎管理、综合保税区扩展贸易功能等试点政策全市共享。

　　积极开展苏州工业园区开放创新综合试验，加快建设中国开发区升级版、世界一流高科技产业园区和国际化开放合作示范区。加强昆山深化两岸产业合作试验区改革创新，重点在产业调整升级、体制机制创新、两岸产业深度融合等方面先行先试。推进太仓中德中小企业合作示范园等载体建设。学习借鉴自由贸易试验区经验，积极复制推广投资、贸易、金融和综合监管制度等方面的改革举措。推进跨境贸易电子商务试点和公共海外仓建设，支持昆山综合保税区开展优化贸易方式、优化监管方式试点。争取常熟服装城、东方丝绸市场获批开展市场采购贸易试点。建成苏州药品口岸检验所，完善张家港汽车整车进口口岸和太仓港启运港退税功能。更加有效地引进境外资金和先进技术，引导外资投向新兴产业和现代服务业。主动投身“一带一路”和长江经济带建设，用好“苏满欧”等国际物流通道，建设太仓港长江水运集散中心，力争苏州货运西站对外开放二类口岸早日获批。支持企业扩大对外投资，开展国际产能合作、境外加工贸易、资源开发和营销网络建设，拓展对外工程承包领域。做好外事、侨务工作，注重发挥国际友城作用，促进经贸合作与人文交流。服务中央对台工作大局，深化与台湾地区的交流合作。

　　4.注重统筹协调，推动城乡一体发展。注重统筹协调，推动城乡一体发展。启动修编苏州市城市总体规划（2030年），制定重点村、特色村规划，编制城市综合交通体系规划，开展中环快速路二期、太湖新城苏州湾东西隧道、独墅湖第二通道等规划研究。推进沪通铁路苏州段建设，开展通苏嘉城际铁路、沿江城际铁路和太仓港铁路支线前期工作。建成常嘉高速公路、张家港疏港高速公路，开工建设锡通过江通道公路南连接线，改扩建312国道苏州东段，启动实施城北路改建、南湖快速路东延工程，续建西环快速路南延、南环快速路西延等工程。实现轨道交通2号线延伸线试运行，加快建设3号线、4号线及支线，开工建设5号线，争取市域S1号线开工建设；建设苏州高新区有轨电车2号线和1号线延伸线。实施太湖新城等地下综合管廊工程，建好海绵城市试点项目。新增人防设施110万平方米。继续整治苏申内港线、外港线和张家港福南水道，做好青阳港航道整治前期工作。建成特高压淮上线工程，实施分布式光伏、智能岸电等示范项目。完善信息基础设施，打造智慧城市。实施市政道路专项大修，推进市区人民路综合整治提升工程。健全城市管理体制机制，充分发挥“数字城管”作用，全面实行网格化管理，提高信息化、精细化、常态化管理水平。

　　深化农村集体产权制度改革，完成土地承包经营权确权登记颁证工作，依法推进土地经营权有序流转。继续开展社区股份合作社股权固化改革，健全农村集体资产管理体系。抓好“政经分离”改革，明确合作经济组织承担的公共服务范围和标准。放大城乡一体化基金效应，提高支农惠农成效。逐步完善农业信贷担保体系和农业保险制度，扩大农民土地承包经营权抵押试点范围。全面实行土地、房屋、林地、滩涂等不动产统一登记，认真开展第三次全国农业普查。扎实开展农村人居环境整治行动，启动新一批美丽城镇建设项目，新增美丽村庄示范点10个、三星级康居乡村300个。

　　5.改善生态环境，建设美丽宜居家园。改善生态环境，建设美丽宜居家园。坚决执行最严格的环境保护制度，统筹抓好污染治理、节能减排、低碳发展、生态修复等工作。深入贯彻新《大气污染防治法》，实施燃煤机组超低排放改造，协同治理工业废气污染；强化机动车船等移动源污染治理，严控挥发性有机物、扬尘和油烟污染，完善重污染天气应对措施，确保PM2.5浓度持续下降。严格保护水源地，保障城乡供水安全。实施水污染防治行动计划，持续开展太湖流域水环境综合治理，推进阳澄湖新三年生态优化行动。全面开展控源截污，提升断面水质达标率。完善河道长效管理机制，建设片区活水工程，完成1000个自然村庄生活污水治理任务。强化噪声污染、核与辐射防治以及危险废弃物管理，抓好土壤环境质量检测及修复治理。实施工业污染源全面达标排放计划，大幅度减少污染物排放。加大农业面源污染防治力度，推进种养业废弃物资源化利用、无害化处置。促进城乡生活垃圾分类投放、收集、运输和处置，构建再生资源回收体系。建设中大型生活垃圾转运站、沿七子山垃圾车专用通道和生活垃圾焚烧发电、餐厨垃圾处置提标改造项目，建成吴江区生活垃圾焚烧发电项目。推行环境监察网格化、执法信息化，加强联动检查执法，严厉打击偷排偷放、数据造假等违法行为。实行最严格的水资源管理和节约用地制度，严控能源、水资源、建设用地总量和强度。全面实施“三优三保”，促进土地节约集约利用。建立节能量交易制度，推广合同能源管理，深入开展重点企业节能低碳行动和“能效之星”创建活动。按照建设国家低碳试点城市的要求，完善碳排放数据管理系统和重点企事业单位温室气体排放报告制度。加大生态支付和生态补偿力度，探索建立生态环境资源界定和评估制度、绿色审计制度。支持绿色清洁生产，推行企业循环化生产、产业循环化组合、园区循环化改造，建设张家港国家再制造产业示范基地和循环经济重点园区、示范企业。推广新能源汽车，鼓励绿色出行。提高建筑节能标准，推广绿色建筑和建材，加快建筑产业现代化。开展反过度包装、反食品浪费、反过度消费行动，推动形成勤俭节约的社会风尚。严守耕地保护、开发强度和生态保护红线，以主体功能区规划为基础统筹各类空间性规划，推进“多规合一”。优化提升“四个百万亩”布局，充分发挥其生态效益。深入实施生态文明建设三年行动计划，继续建设上方山石湖生态园、虎丘湿地公园等重点项目，办好第九届江苏省园艺博览会。开展植树造林，完成森林抚育6.67千公顷，市区新增绿地350万平方米。加大湿地保护力度，扩大湿地保护范围，保护湿地生态空间。

　　6.繁荣各项事业，促进社会和谐进步。繁荣各项事业，促进社会和谐进步。坚持普惠性、保基本、均等化、可持续方向，从解决人民群众最关心、最直接、最现实的利益问题入手，提高公共服务共建能力和共享水平。深化教育领域综合改革，提升教育质量，促进教育公平。实施学前教育新五年行动计划，大力发展普惠性幼儿园。加快义务教育优质均衡发展，推动普通高中多元特色发展。落实外来务工人员子女学校教育质量提升计划，启动民办学校“校安工程”。加强素质教育，增强学生社会责任感、创新精神、实践能力。实施中小学家庭教育课程项目。深化产教融合、校企合作，促进职业教育改革发展。推进名城名校融合发展，提高在苏高校教学水平和创新能力，更好服务地方发展。坚持医疗、医保、医药联动，做好医药卫生体制改革试点工作。建立分级诊疗制度，发展“医联体”，完善医保支付政策，加快形成基层首诊、双向转诊、急慢分治、上下联动的就医秩序。启动健康苏州2020战略，逐步建立社区健康管理中心和家庭医生护士执业管理新机制。出台新一轮医疗机构设置规划，续建市公共医疗中心、第九人民医院等医疗机构，构建城市多中心疾病协同救治体系。全面实施一对夫妇可生育两个孩子政策。健全现代公共文化服务体系，建设苏州第二图书馆、第二工人文化宫、历史文化陈列馆、考古博物馆、桃花坞木刻年画博物馆、方志馆新馆和吴中区公共文化中心。倡导全民阅读，建设“书香城市”。创建国家级文艺创作基地，扶持优秀文化产品创作生产，鼓励苏州文化“走出去”。加强历史文化名城名镇名村和各类文化遗产保护，做好江南水乡古镇和海上丝绸之路申报世界文化遗产工作。完成地方专业志编撰工作。推进苏州工业园区体育中心、运河体育主题公园、市民健身步道和江苏省苏州体育训练基地等体育设施建设。办好“汤尤杯”世界羽毛球团体赛、第14届市运会等赛事。做好民族宗教、妇女儿童工作。加强国防动员和民兵预备役建设，扎实开展拥军优属工作，认真落实优抚安置政策，推动军民深度融合发展。

　　坚持用邓小平理论、“三个代表”重要思想、科学发展观和习近平总书记系列重要讲话精神武装头脑、教育人民，用中国梦和社会主义核心价值观凝聚共识、汇聚力量。建立长效常态机制，深化文明城市建设。繁荣发展哲学社会科学，建设地方特色新型智库。做好科学普及工作，提高全民科学素质。加强城乡社区协商民主建设，开展社区治理现代化试点，建成集管理服务、民意沟通、信息公开于一体的社区信息平台一期项目。指导社区居委会、村委会换届选举。推进政社分开，规范和引导社会组织健康发展。建设公共信用基础数据库和服务平台二期项目，建立健全社会信用体系。把安全放在城市发展的第一位，强化“红线”意识和“底线”思维，坚决执行党政同责、一岗双责、失职追责各项规定，完善和落实安全生产责任和管理制度，切实做到市、县级市（区）、镇（街道、开发区）和行政村全覆盖。严格督促企业落实主体责任，建立覆盖全员的安全生产责任制。坚持重大隐患三级政府挂牌督办制度，深化重点领域专项整治，着力消除安全事故隐患，坚决杜绝重特大事故。加强信访法治化建设，通过法定途径分类处理信访投诉请求，推行“阳光信访”，及时回应和解决群众合理诉求。坚持重大决策风险评估制度，完善社会矛盾排查预警和调处化解综合机制。发展专业性、行业性人民调解组织，健全人民调解、行政调解、司法调解联动工作体系。制定实施“七五”普法规划，加强公共法律服务，提高法治惠民实效。完善立体化现代化社会治安防控体系，推动警务改革，积极防范、依法惩治违法犯罪活动，维护社会秩序。贯彻总体国家安全观，依法严密防范和严厉打击敌对势力渗透颠覆破坏活动、暴力恐怖活动、民族分裂活动、极端宗教活动。全面推进法治苏州建设，积极争创全省、全国法治城市创建先进市。强化应急管理体系建设，提升突发公共事件应急处置能力。建设灾害避难场所，加强救灾物资储备，创建全国综合减灾示范社区。

　　7.落实惠民措施，增进人民群众福祉。落实惠民措施，增进人民群众福祉。提高民生支出占财政支出的比重，优化民生保障体系，办好各项民生实事。实施就业优先战略和更加积极的就业政策，全年新增就业15万人，苏州籍应届高校毕业生就业率保持在95%以上。实施创业天使计划，发展众创空间。维护职工合法权益，构建和谐劳动关系。落实全民参保计划，基本实现法定人员全覆盖。开展机关事业单位养老保险制度改革，完善社会医疗保险管理办法。聚焦低收入家庭和经济薄弱村，强化精准帮扶、精准脱困措施，引导全社会以多种形式参与扶贫帮困。健全“救急难”工作机制，推动临时救助对象由户籍人口向常住人口拓展。全面实施城乡居民大病保险制度，探索建立老年人大病长期护理保险制度。加快医养融合发展，推进公办养老机构改革。健全以居家养老为基础、社区为依托、机构为补充的养老服务体系，虚拟养老院服务覆盖所有村和社区。完善残疾人社会保障制度，扩大政府购买助残服务范围，增强社区助残服务能力。支持慈善事业发展，动员社会力量开展救济救助和志愿服务。建设保障性安居工程，加大住房保障货币化补贴力度。落实棚户区改造任务，继续实施梅巷片区、解放新村危旧房改造项目。扩大住房公积金覆盖面，提高职工缴存水平。实施流动人口积分管理，优化流动人口服务。建成苏州汽车西站综合客运枢纽和城市公共交通智能化应用示范工程，市区新购公交车640辆，新辟及优化常规公交线路50条。建好12345政府公共服务平台国家级标准化试点项目。深入开展食品安全城市创建活动和食品药品安全放心行动，积极构建严密高效、社会共治的食品药品安全治理体系。落实粮食安全责任制，建设“粮安工程”。加大“菜篮子”基地建设力度，提高农产品自给水平。健全市场价格监测预警体系，及时防范和应对价格异常波动。

　　8.加强自身建设，提高政府服务效能。加强自身建设，提高政府服务效能。加快建设法治政府和服务型政府，努力为人民群众提供优质高效服务。严守政治纪律和政治规矩，自觉维护党中央权威，确保政令畅通。着力践行“三严三实”要求，始终做到忠诚、干净、担当。不折不扣贯彻落实关于改进工作作风、密切联系群众的各项规定，坚持不懈反对“四风”。深化领导干部下基层“三访三促”活动，虚心听取群众意见和建议，切实改进工作方式方法，主动为基层、为企业、为群众排忧解难。牢固树立并不断强化清醒、理性、务实的工作导向，对确定的目标、制定的政策、部署的任务，一抓到底，务求实效。严格遵守宪法和法律，按照法定权限和法定程序行使权力、履行职责。高质量起草地方性法规议案，制定政府规章和规范性文件。强化执法责任，优化程序规定，细化操作流程，坚持严格规范公正文明执法。推进行政执法体制改革，继续实施部门内部综合执法试点，探索跨部门、跨行业综合执法和区域综合执法。健全行政复议、行政调解制度，做好行政复议案件办理、行政诉讼案件出庭应诉等工作。完善政务服务管理办公室与政务服务中心、便民服务中心、公共资源交易中心一体化运行管理模式，完成工程建设项目招投标、土地使用权和矿业权出让、国有产权交易、政府采购等交易平台整合工作，做到规则统一、公开透明、服务高效、监督规范。优化企业投资项目并联审批和“多评合一”、“多图联审”，开辟重点项目和民生工程审批“绿色通道”。加强机关部门绩效管理，提高履职能力和服务水平。厉行勤俭节约，反对奢侈浪费，严控“三公”经费支出。严格落实党风廉政建设责任制，切实履行“一岗双责”，坚决执行《中国共产党廉洁自律准则》和《中国共产党纪律处分条例》，深入推进惩治和预防腐败体系建设，始终保持惩治腐败高压态势。加强对公共资金、国有资产、国有资源和领导干部经济责任履行情况的审计，抓好对重大政策措施落实情况的跟踪审计，探索开展领导干部自然资源资产离任审计。做好政府信息公开工作，及时回应公众关切。各级政府要自觉接受人民代表大会及其常委会的法律监督、工作监督，接受人民政协的民主监督，接受司法机关的监督，接受新闻舆论和社会公众的监督，认真实施人大议案，办理代表建议和政协提案，不断提高政府工作水平。

　　各位代表，“十三五”发展的新征程已经开启，我们肩负的使命重大而光荣、任务繁重而艰巨。让我们更加紧密地团结在以习近平同志为总书记的党中央周围，深入贯彻落实党的十八大和十八届三中、四中、五中全会精神，在中共苏州市委的坚强领导下，团结一心，开拓进取，为“十三五”发展取得良好开局、率先全面建成小康社会、谱写好中华民族伟大复兴中国梦的苏州篇章而努力奋斗！
